# Successful Rituximab Therapy for Skin Sclerosis and Myositis in a Patient With Systemic Sclerosis, Myositis and Sjögren’s Syndrome Associated With Autoimmune Polyendocrine Syndrome Type 2

**DOI:** 10.7759/cureus.45831

**Published:** 2023-09-23

**Authors:** Takako Saeki, Hiroki Nishiyama, Haruna Kimura, Hiroyuki Usuda, Kazuo Furukawa

**Affiliations:** 1 Internal Medicine, Nagaoka Red Cross Hospital, Nagaoka, JPN; 2 Dermatology, Nagaoka Red Cross Hospital, Nagaoka, JPN; 3 Pathology, Nagaoka Red Cross Hospital, Nagaoka, JPN

**Keywords:** type i diabetes mellitus, autoimmune thyroid disease, rituximab therapy, inflammatory myositis, diffuse systemic sclerosis, autoimmune polyendocrine syndrome type ii

## Abstract

Autoimmune polyendocrine (or polyglandular) syndrome (APS) is a relatively rare clinical condition characterized by functional impairment of multiple endocrine glands due to loss of immune tolerance. APS is broadly categorized as rare monogenic forms, such as autoimmune polyendocrine syndrome type 1 (APS-1), and a more common polygenic variety, autoimmune polyendocrine syndrome type 2 (APS-2). Although many autoimmune conditions including autoimmune rheumatic diseases can develop in APS-2, systemic sclerosis or myositis as a complication is quite rare and no treatment strategy has yet been established.

A 25-year-old man who had been diagnosed as having type 1 diabetes developed finger stiffness. Although the subjective symptoms were relatively mild, extensive examinations including various autoantibodies, hormones and biopsy of the skin and minor salivary glands revealed that he had APS-2 (type 1 diabetes and autoimmune thyroid disease) accompanied by systemic sclerosis, myositis and Sjögren’s syndrome. Rituximab therapy was initiated for the progressive skin sclerosis, and this resulted in significant alleviation of both the sclerosis and the myositis. In APS, early diagnosis and immunomodulatory therapy may arrest the autoimmune process before irreversible organ damage has occurred. This case report suggests that rituximab may be a promising therapy for autoimmune rheumatic diseases associated with APS-2.

## Introduction

Autoimmune polyendocrine (or polyglandular) syndrome (APS) comprises a diverse group of clinical conditions characterized by functional impairment of multiple endocrine glands due to loss of immune tolerance [1]. The syndrome can be broadly categorized as rare monogenic forms, such as autoimmune polyendocrine syndrome type 1 (APS-1), and a more common polygenic variety, autoimmune polyendocrine syndrome type 2 (APS-2) [1]. APS-1 is a rare autosomal recessive disease caused by mutations in the autoimmune regulator gene, and is characterized by the development of at least two of three cardinal components during childhood: chronic mucocutaneous candidiasis, hypoparathyroidism, and primary adrenal insufficiency (Addison’s disease). On the other hand, APS-2 can show courses characterized by at least two of three endocrinopathies: type 1 diabetes, autoimmune thyroid disease and Addison’s disease. Although some authors have proposed splitting this syndrome into APS-2, APS-3 or APS-4 [2], the broader term APS-2 is now considered appropriate for all affected patients because there is little evidence for distinct causes in such subcategories [1]. The prevalence of APS-1 and APS-2 is 1:100000 and 1:1000, respectively, and APS-2 is a milder and later-onset disease. In APS-2, many autoimmune conditions can develop, including celiac disease, alopecia, vitiligo, primary ovarian insufficiency, pernicious anemia and various autoimmune rheumatic diseases.

Here we present a rare case of systemic sclerosis, myositis and Sjögren’s syndrome associated with APS-2 for which rituximab therapy was quite effective for control of progressive skin thickening and muscle lesions.

Previously posted server: The first version of this article was previously posted to the Research Square preprint server on August 3, 2023.

## Case presentation

A 25-year-old Japanese male was referred to our hospital because of finger stiffness and body weight loss (-7 kg over three months). Three years previously, he had been diagnosed as having latent adult autoimmune (type 1) diabetes on the basis of a random plasma glucose level of 284 mg/dl, a hemoglobin A1c (HbA1c) level of 6.3%, and a markedly elevated serum anti-glutamic acid decarboxylase (GAD) antibody level (1143.3 U/ml, normal <5.0). He had been treated with voglibose 0.6 mg daily and his HbA1c level was 5.7%. He had no family history of diabetes, thyroid disease or rheumatic disease. His height, body weight, blood pressure and pulse rate were 175 cm, 55 kg, 132/83 mmHg and 118/min, respectively. On clinical examination, the proximal interphalangeal (PIP) joints were slightly swollen and tender. Although Raynaud’s phenomenon was absent, there was skin thickening on the fingers of both hands extending proximally to the metacarpophalangeal joints and anterior chest [modified Rodnan total skin thickness score (mRSS) 5 points at the first visit], and telangiectasia was evident on the anterior chest. Mild myalgia and muscle tenderness were evident on the bilateral thighs, without apparent muscle weakness. There were no other abnormal findings.

Laboratory data (Table 1) showed high serum levels of creatine kinase (CK) (1198 U/l) (normal: 59-248), aspartate aminotransferase (AST) 58 U/l (13-30), alanine aminotransferase (ALT) 58 U/l (10-42), and lactate dehydrogenase (LDH) 265 U/l (124-222). The serum C-reactive protein level was 0.05 mg/dl; IgG was 2111 mg/dl (861-1747), IgA was 327 mg/dl (93-393), and IgM was 64 mg/dl (33-183). Electrocytes, as well as urinalysis and renal function parameters, were all within the normal ranges. Rheumatoid factor and anti-CCP antibody were negative. Anti-nuclear antibody (ANA) was positive (x2560) with a nucleolar and speckled pattern, and only anti-Ro/SSA was positive (149 U/ml, normal <9.9) among disease-specific autoantibodies including scleroderma or myositis-related autoantibodies. On the basis of the levels of thyroid-stimulating hormone (TSH) 0.01 mIU/ml (normal: 0.35-4.94), free triiodothyronine (FT3) 15.90 pg/ml (1.68-3.67), free thyroxine (FT4) 3.98 ng/dl (0.7-1.48) and TSH receptor antibody (TRAb) 21.6 IU/l (normal <2.01), the patient was diagnosed as having Graves’ disease. Anti-thyroid peroxidase (TPO) antibody and anti-thyroglobulin antibody were also positive (576 IU/ml, normal <5.61, and 118.0 IU/ml, normal <27, respectively). Adrenocortical parameters were normal.

**Table 1:** Laboratory findings WBC: white blood cells; RBC: red blood cells; Hb: haemoglobin; Plt: platelets; AST: aspartate aminotransferase; ALT: alanine aminotransferase; LDH: lactate dehydrogenase; ALP: alkaline phosphatase; CK: creatine kinase; BUN: blood urea nitrogen; Cre: Creatinine; CRP: C-reactive protein; ANA: Anti-nuclear antibody; TSH: thyroid-stimulating hormone; CCP: cyclic citrullinated peptide; FT3: free triiodothyronine; FT4: free thyroxine; TRAb: TSH receptor antibody; TPO: thyroid peroxidase; GAD: glutamic acid decarboxylase.

Hematology			Serology					
WBC	4600 (3300-8600)	/µL	CRP	0.05 (0-0.14)	mg/dL	Anti-GAD	1143 (<0.5)	U/mL
RBC	498 (435-555)x 10^4^	/µL	IgG	2111 (861-1747)	mg/dL	Anti-TPO	576 (<5.61)	IU/mL
Hb	13.3 (13.7-16.8)	g/dL	IgA	327 (93-393)	mg/dL	Anti-Tg	118 (<27)	IU/mL
Plt	19.1(15.8-34.8)x 10^4^	/µL	IgM	64 (33-183)	mg/dL	TRAb	21.6 (<2.01)	IU/L
			CH50	59 (32-57)	U/mL			
Biochemistry								
TP	7.6 (6.6-8.1)	g/dL	Autoantibody			Hormones		
Alb	4.0 (4.1-5.1)	g/dL	RF	Negative		FT3	15.90 (1.68-3.67)	pg/mL
AST	58 (13-31)	U/L	Anti-CCP	Negative		FT4	3.98 (0.7-1.48)	ng/dL
ALT	58 (10-42)	U/L	ANA	x 2560	(Sp+Nuc)	TSH	0.01 (0.35-4.94)	mIU/mL
ALP	175 (38-113)	U/L	Anti-SSA	149 (<9.9)	U/mL			
LDH	265 (124-222)	U/L	Anti-SSB	Negative		Cortisol	12.8 (2.9-19.4)	μg/dL
CK	1198 (59-248)	U/L	Anti-dsDNA	Negative		ACTH	29.0 (7.2-63.3)	pg/mL
BUN	17.2 (8-20)	mg/dL	Anti-Sm	Negative				
Cre	0.35 (0.65-1.07)	mg/dL	Anti-RNP	Negative				
Glucose	90 (73-109)	mg/dL	Anti-Scl70	Negative				
HbA1c	5.9 (4.9-6)	%	Anti-RNA polymerase III	Negative				
			Anti-centromere	Negative				
Urinalysis			Anti-ARS	Negative				
Protein	(-)		Anti-MDA5	Negative				
Occult blood	(-)		Anti-TIF1γ	Negative				
Sugar	(-)		Anti-M2	Negative				

**Figure 1:**
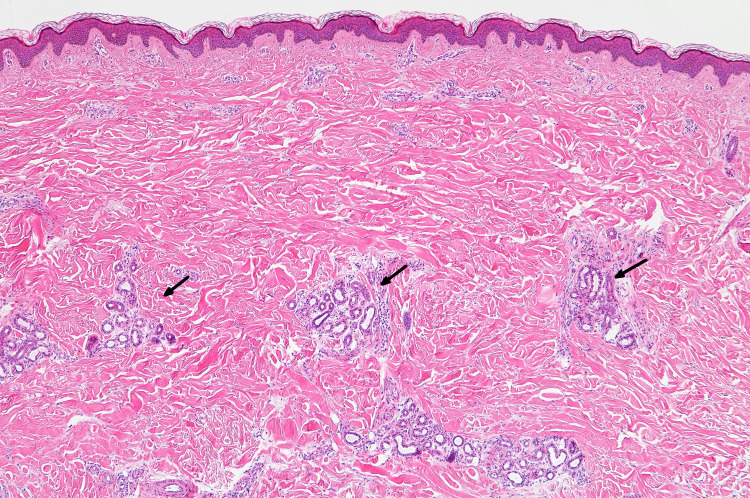
Light microscopy image of a forearm skin biopsy. Atrophic eccrine glands entrapped within thickened dermal collagen are demonstrated (Hematoxylin and eosin stain, ×10).

A forearm skin biopsy revealed atrophic eccrine glands entrapped within thickened dermal collagen, compatible with scleroderma (Figure 1).

**Figure 2:**
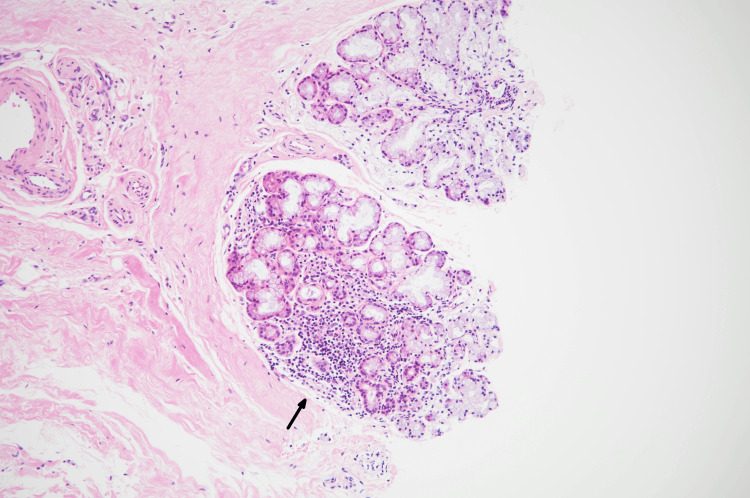
Light microscopy image of a minor salivary gland biopsied from the lip. Infiltration of lymphocytes around the salivary gland is evident (Hematoxylin and eosin stain, ×25).

Although subjective dry eyes and dry mouth were absent, Schirmer tests (right; 6 mm/5 min; left; 4 mm/5 min) were positive and biopsy of a minor salivary gland revealed periductal infiltration of lymphocytes (focus score 1) (Figure 2).

**Figure 3:**
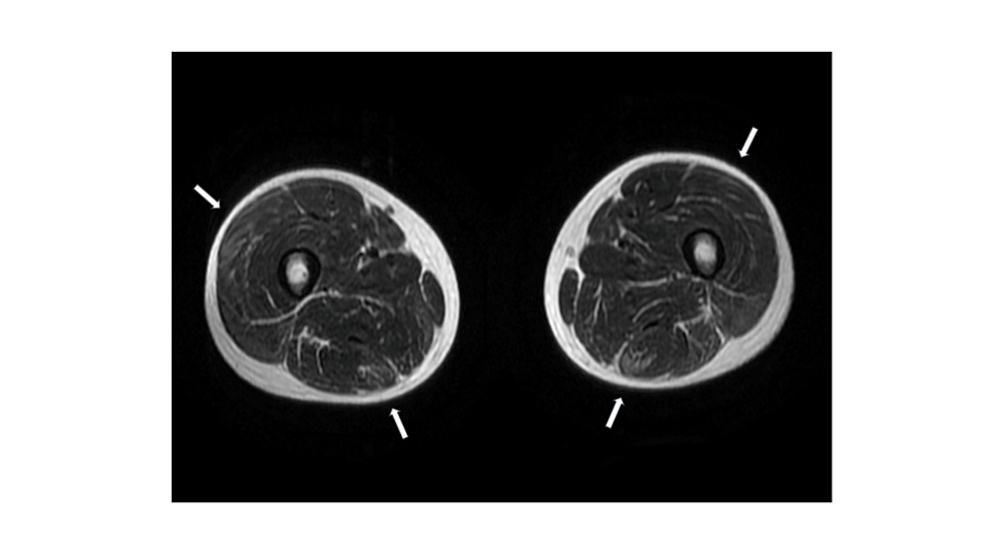
Magnetic resonance imaging of both thighs. Axial T2-weighted image reveals bilateral patchy hyperintense lesions in both muscles.

Axial T2-weighted magnetic resonance imaging (MRI) of both thighs revealed bilateral patchy hyperintense lesions, consistent with myositis (Figure 3).

On the basis of these results, the patient was diagnosed as having systemic sclerosis, Sjögren’s syndrome and myositis associated with APS-2 (type 1 diabetes and autoimmune thyroid disease). Further examinations revealed no evidence of any interstitial lung disease, or heart, renal and gastrointestinal lesions. Thiamazole was added to the voglibose, and the patient’s thyroid function gradually improved. However, the skin thickening rapidly worsened. Two months after the first visit, the mRSS had increased to 13 points (bilateral fingers 2 in each, bilateral forearms 3 in each, and anterior chest 3). Although dyspnea was absent, pulmonary function tests revealed a decreased vital capacity (VC) (% VC 62.3%). Diffusing capacity (DLCO) was normal and high-resolution computed tomography demonstrated no interstitial lung lesions, suggesting that the decreased VC was due to sclerosis of the anterior chest skin.

Rituximab (RTX) was then administered at a dose of 375 mg/m^2^ once a week for four weeks. During the initial three months after RTX therapy, there were no significant changes in the skin thickness (mRSS 12 points) or the level of serum CK (1605 U/l). However, at six months after completion of the RTX therapy, the mRSS had decreased to 7 points (bilateral fingers 1 in each, bilateral forearms 2 in each, and anterior chest 1). VC was slightly increased (% VC 66.5%) and the level of CK was decreased (725 U/l). At six months after the initial RTX therapy, we administered additional RTX at a dose of 375 mg/m^2^ once a week for two weeks and methotrexate was administered as maintenance therapy. At one year after the first RTX therapy, the mRSS was 4 points (bilateral fingers 1 in each, bilateral forearms 1 in each, and anterior chest 0), % VC was 73.5%, and the level of CK was within the normal range (79 U/l).

## Discussion

This report describes a rare case of APS-2 accompanied by systemic sclerosis, myositis and Sjögren’s syndrome as non-endocrine autoimmune disorders. Sjögren’s syndrome has sometimes been reportedly associated with APS-2 [3], although the precise frequency has remained unclear. However, systemic sclerosis or myositis associated with APS-2 is quite rare.

In general, management of APS includes hormone replacement therapy as needed and treatment of complications [1]. In the present case, symptoms associated with type 1 diabetes and Graves’ disease were mild and both conditions had been well controlled with non-immunosuppressive therapy. On the other hand, progressive skin sclerosis on the anterior chest was problematic because it had led to a decrease of VC.

RTX is a chimeric antibody against CD20, a cell membrane molecule specifically expressed in B cells. Although the pathogenesis of systemic sclerosis remains unclear, B cells are thought to play a central role, and B-cell depletion therapy with RTX has been attempted [4]. In Japan, the DESIRES study was conducted from 2017 to 2019 involving 56 Japanese patients with systemic sclerosis [5]. In this double-blind, placebo-controlled trial with a primary-endpoint measure of skin sclerosis, RTX demonstrated significant efficacy compared to placebo and RTX was approved for treatment of systemic sclerosis in Japan in 2021. Although it has still not been approved in the United States and Europe because the DESIRES trial involved only Japanese patients, RTX has been reported to significantly improve mRSS relative to respective control groups in some controlled studies conducted in Europe and India [4], suggesting that its efficacy may not be restricted to Japanese patients.

In the present case of APS-2, skin sclerosis and CK elevation were significantly improved with RTX, suggesting a significant role of B cells in APS-2 and associated non-endocrine autoimmune disorders. In fact, RTX has been reported to have beneficial effects on pneumonitis and malabsorption associated with APS-1 [1], although the number of cases has been limited so far.

APS is insidious, being characterized by circulating autoantibodies and lymphocytic infiltration of affected tissues or organs, eventually leading to organ failure. The onset of APS-2 typically occurs in young adulthood, but diagnosis is difficult during the asymptomatic phase because there are no specific tests for the detection of APS-2. However, testing for autoantibodies may be helpful for the assessment of disease risk, as it has been reported that the relevant autoantibodies are frequently detectable years before disease onset [1]. Although APS is rare, physicians should be aware of this condition and consider testing for various autoantibodies in young adult patients diagnosed as having autoimmune endocrine diseases. Physicians should also be aware that APS-2 carries an increased risk for the development of other autoimmune diseases. In fact, in the present case, extensive examinations revealed various concurrent autoimmune diseases, even though subjective symptoms were mild. In APS, early diagnosis and immunomodulatory therapy may arrest the autoimmune process before irreversible organ damage has occurred. This case report suggests that rituximab may be a promising therapy for autoimmune rheumatic diseases associated with APS-2. Further accumulation of cases is necessary.

## Conclusions

Although APS is a relatively rare condition, physicians should be aware that various autoimmune diseases without apparent subjective symptoms can develop as complications. Early diagnosis and immunomodulatory therapy may make it possible to arrest the autoimmune process before irreversible organ damage has occurred, and RTX appears to be a promising therapy for autoimmune rheumatic diseases associated with APS-2. Further accumulation of cases is necessary.
